# Mitral valve surgery using video-assisted right minithoracotomy and deep hypothermic perfusion in patients with previous cardiac operations

**DOI:** 10.1186/s13019-015-0259-0

**Published:** 2015-04-17

**Authors:** H Tarık Kızıltan, Aslı İdem, Salih Salihi, Ali Soner Demir, Aşkın Ali Korkmaz, Mustafa Güden

**Affiliations:** 1 Cardiovascular Surgery, Özel Adana Hastanesi, Hekimköy Sitesi Sarıçam D-5 No:108, 01000 Adana, Turkey; 2 Anesthesiology and Reanimation, Özel Adana Hastanesi, Adana, Turkey; 3 Cardiovascular Surgery, Fatih University, Medical Faculty, Istanbul, Turkey; 4 Cardiology, Fatih University, Medical Faculty, İstanbul, Turkey

**Keywords:** Cardiopulmonary bypass, Hypothermia/circulatory arrest, Mitral valve, Reoperation

## Abstract

**Background:**

Redo-sternotomy for mitral valve (MV) surgery may be complex and attendant complications can be avoided using anterolateral right thoracotomy, deep hypothermia (20°C, nasopharyngeal) with low flow cardiopulmonary perfusion. Video-assisted minithoracotomy technique is a further improvement.

**Methods:**

We performed 20 consecutive MV operations in patients with previous cardiac surgery using video-assisted right minithoracotomy, femoro-femoral bypass, deep hypothermia, low flow cardiopulmonary bypass without aortic cross-clamping. The mean follow-up was 30 ± 17.8 mo. Data is presented as the mean ± standard deviation of the mean.

**Results:**

There were 11 males and 9 females (age, 62.3 ± 12.1; ejection fraction 50.1 ± 11.2). Operations included MV replacement (n = 11), MV repair (n = 5), and MV re-replacement (n = 4). There were no hospital deaths, and the mean hospital stay was 8 ± 2.9 days. There were no postoperative strokes or need for mechanical circulatory support. The mean cardiopulmonary bypass time was 152 ± 28 minutes. Two patients (10%) required inotropic support beyond 24 hrs. All patients were free from inotropic support at 48 hours. The mean number of transfused red cell units was 2.8 ± 0.8 (range, 2 to 4). One patient died in another institution six months postoperatively following surgery for acute type III aortic dissection. At 30 ± 17.8 months follow-up all patients were found to be in NYHA Class I or II.

**Conclusions:**

Minimally invasive video-assisted MV surgery using deep hypothermia, low-flow cardiopulmonary bypass without aortic clamping can result in excellent clinical outcomes in patients with previous cardiac surgery via a median sternotomy. This technique offers reproducible results, good myocardial protection (as evidenced by the low rate of inotropic support that patients needed postoperatively), and low rates of complications.

## Background

In patients with previous cardiac operations, there are well-known risks of repeat median sternotomy, including excessive bleeding, mediastinitis, cardiac tamponade, dehiscence, sternal osteomyelitis, and injury to cardiac structures and/or patent coronary grafts [1,2]. These complications mostly relate to the surgical trauma to the densely healed sternum and dissection of the intense adhesions surrounding the heart and coronary arteries, with or without previous bypass grafts. As an alternative surgical strategy — which mostly eliminated the above risks — right thoracotomy, femoro-femoral bypass, and deep hypothermia with low flow perfusion were introduced into clinical practice as a means of approaching the mitral valve (MV) during reoperative surgery [3-5]. Many utilized right thoracotomy in reoperative MV procedures with favorable results [6-8]. A video-assisted minithoracotomy technique has also been applied in this context as a means of less invasive surgery [8-13].

With the premise of less tissue damage and improved results on a difficult group of patients, we embarked on a program of performing MV surgery using video-assisted right minithoracotomy, deep hypothermia, low flow cardiopulmonary bypass (CPB), and no-aortic clamping in patients with prior cardiac operations. We aimed to report our indications, technique, and results on this contemporary management strategy.

## Methods

Over the years, our group has performed video-assisted minimally invasive MV surgery on more than 300 patients [14]. This study concerns a consecutive group of 20 of those 300 patients, all 20 of whom had prior sternotomies and subsequently underwent the reoperative MV procedure between August 2009 and December 2013 using the same technique. The study was approved by the institutional review boards of our hospitals and an informed consent was obtained from all patients. Included in our study were all patients undergoing isolated mitral or mitral/tricuspid valve surgery with trivial or less aortic insufficiency. All patients underwent a computerized tomography (CT) to ascertain risk of CPB via femoral cannulation and rule out aortoiliac aneurysm with thrombus formation. One patient with known descending thoracic aneurysm underwent CT angiography to exclude aortic dissection.

Three surgeons performed the operations in this study while at least two of them actively participated for each case. After induction of general anesthesia and single lumen endotracheal intubation, a slight lateral decubitus position was given to the patients. In all patients, a transesophageal echocardiography (TEE) probe was inserted at this stage of the operation and TEE was performed to evaluate overall cardiac and valvular functions. In particular, the feasibility of MV repair was explored [15]. Our surgical technique included a small (5 cm) right fourth intercostal space incision and two thoracic ports at the anterior axillary line for the video camera, cardiotomy vent, and CO_2_ insufflation ― 2 L/min ― catheter. Intercostal muscles longer than the corresponding skin incision were dissected. A specially designed rib spreader (Estech, San Ramon, California) and a left atrial retractor (with a supporting arm) passing through the thoracic wall were used. A small (4 cm) femoral fold incision was performed. Direct arterial cannulation method ― femoral artery snared over the cannula at proximal and clamped at distal part ― was utilized in all patients. A femoral arterial (17 Fr, Medtronic, MN, USA) and a long venous cannula (30/33 Fr, Medtronic) were inserted for femoro-femoral CPB. Additionally, a transcutaneous internal jugular venous cannula (17 Fr, Medtronic, MN, USA) (n = 3) and a separate superior vena cava cannula (n = 1) were utilized for satisfactory venous return. Vena cavae were snared (n = 2) in patients with planned tricuspid valve repair. During CPB, patients were cooled down to 20°C. The pericardium was opened 2 cm above the phrenic nerve and a left atrial incision was performed between right pulmonary veins and interatrial groove. The CPB flow was reduced as the hypothermia progressed, and exposure of the MV succeeded accordingly. On CPB, patients developed ventricular fibrillation spontaneously. Once 20°C was reached, excepting infrequent periods of very low CPB perfusion flow (near circulatory arrest) so as to improve MV visualization, minimal CPB flow was 1.5 liter/min. As a general rule, blood pressure above 70 to 80 mmHg was maintained during CPB. Dysfunctional native (n = 11) or prosthetic valves (n = 4) were explanted and prosthetic valves implanted using interrupted mattress sutures. In 8 patients (7 ischemic MI and 1 AVSD patient) undergoing MV replacement (MVR), the posterior MV and chordae were spared. In 3 patients with ischemic MI who also had mitral stenosis, non-chordal sparing MVR was performed. 2 of the 4 patients undergoing MV re-replacement had their posterior MV and chordae intact, and these were spared, too. Five patients received MV repair (chordal replacement, n = 1) with an annuloplasty (downsizing) ring (n = 5). Rewarming was started. The right atrium was opened with the vena cavae snared in two patients and tricuspid repair with an annuloplasty ring was performed. The right atrium was closed. Under increased CO_2_ insufflation ― 8 L/min ―, an 8 Fr venting catheter was introduced into the left ventricle through the valve, the patient was given a slight Trendelenburg and leftward position, and air was meticulously evacuated. The left atrium was closed, rewarming was completed, and the heart was defibrillated using externally placed electrodes. The patient was placed on partial CPB with a left ventricular catheter in place, as previously described [5] to keep one of the mitral prosthetic leaflets incompetent. This methodology allowed air and possible debris to be evacuated through the vent without entering into the systemic circulation. The venting catheter was removed, and patients were then weaned from CPB only after ensuring the absence of left ventricular air at the TEE. Right ventricular pacing wires were placed. Chest tubes were inserted in the right side and inside the pericardium, and the chest was closed. Postoperatively, patients underwent at least one pre-discharge echocardiography which was also performed every 6 mo. after discharge.

The patients’ hospital charts were reviewed and a recent follow-up which included echocardiographic evaluation of the valvular function was obtained. Data is presented as the mean ± standard deviation of the mean.

## Results

The demographic and preoperative characteristics of the patients are summarized in Table 1. The main indication for surgery was congestive heart failure. All patients were in New York Heart Association (NYHA) Class III or IV. Severe disabling hemolytic anemia and congestive heart failure with ascites due to severe paravalvular leak were found in one patient who had undergone MVR twice. Previous operations included partial atrioventricular septal defect (AVSD) repair (n = 1), coronary artery bypass grafting (CABG) and aortic valve replacement (AVR) (n = 1), CABG and MVR (n = 1), MVR (n = 3), and CABG (n = 14). All patients with previous CABG operations (n = 16) had functioning internal thoracic artery (ITA) grafts. We did not have patent ITAs intimately stuck to the posterior sternal table in this series of patients which would constitute an absolute anatomical contraindication to a redo sternotomy. In seven patients there was a patent graft to the right coronary artery originating from the aorta which would have been at risk of damage.

**Table 1:** **Demographic and preoperative patient characteristics**

Variable	Mean ± SD or no
Age	62 ± 12 (28–79) years
Sex	11 male, 9 female
Functional class	
NYHA class III	12
NYHA class IV	8
EF	50 ± 11
EF ≥ 50%	13
EF < 50%	7
MI	13
MS	1
MI and MS	2
Paravalvular leak	2
Bioprosthetic insufficiency	2
Previous operation	
Isolated CABG	14
CABG and AVR	1
CABG and bioprosthetic MVR	1
MVR (mechanical)	2
MVR (bioprosthetic)	1
Partial AVSD repair	1

NYHA: New York Heart Association; EF: Ejection fraction; MI: Mitral valve Insufficiency; MS: Mitral valve stenosis; CABG: Coronary artery bypass grafting; AVR: Aortic valve replacement; MVR: Mitral valve replacement: AVSD: Atrioventricular septal defect.

Operative and postoperative data are summarized in Table 2. The indications for MV surgery were ischemic MV insufficiency (n = 12, all with annular dilatation, 11 of which patients had restricted posterior leaflet motion, while the remaining patient had elongated papillary muscles), paravalvular leak in a previously implanted mechanical prosthesis (n = 2), bioprosthetic MV insufficiency (n = 2), rheumatic mitral stenosis (n = 3) and a mitral cleft causing valvular insufficiency in a partial AVSD (n = 1). Exposure of the MV was satisfactory in all cases — though the MV position was slightly deeper in the thoracic cavity — and no patient required a conversion to a median sternotomy. MVR (n = 15) and repair (n = 5) were performed. All patients survived the operation. The mean CPB time was 152 ± 28 minutes (range, 112 to 200). There were three patients with atrial fibrillation. Ablation surgery for atrial fibrillation was not considered for two reasons for these patients: reoperative surgery and left atrial diameter > 5.5 cm. Five patients ― 3 MVR (1 also having TP), 2 MV re-replacement; mean EF 46%; all having non-chordal sparing MVR ― required a low level of inotropic support in the first 24 hours postoperatively. Two of these continued receiving the support beyond that period. All five were completely weaned off the inotropes at 48 hours. None of these patients required pulmonary artery catheter monitoring to assess cardiac output and/or immediate postoperative echocardiography. On the fifth postoperative day, a 78-year-old woman with peripheral arterial disease and a calcified femoral artery developed femoral artery thrombosis at the femoral artery incision. This patient had also undergone a direct femoral artery cannulation, as had all other patients in this series. A thromboembolectomy was performed and, after excising the 4 cm portion of the artery, an 8 mm Dacron graft was interposed in the femoral artery due to localized dissection and poor arterial quality. She also developed groin infection, and required a local surgery, and then recovered uneventfully. Another patient developed femoral hematoma seven days postoperatively and required evacuation of the hematoma. One patient developed right diaphragmatic elevation on CXR postoperatively; the patient has been clinically asymptomatic. No other complications such as stroke, low cardiac output, myocardial infarction, prosthetic valve endocarditis, pleural effusion, pneumothorax or pneumonia have been observed. Preoperative screening using CT demonstrated 5.3 cm descending thoracic aneurysm without thrombus formation in a 63-year-old patient who had previously undergone CABG. Having patent four-vessel bypass grafts with functioning ITA, this patient underwent a successful MVR utilizing femoral artery cannulation without experiencing postoperative neurologic dysfunction. We did not consider concomitant or endovascular aneurysm repair shortly after his operation before his aneurysm diameter reached 5.5 cm. The follow-up showed that he died in another institution six months postoperatively following surgery for acute type III aortic dissection. All patients had echocardiographic evaluation showing good valvular function and the patients receiving mechanical valves were anticoagulated using Warfarin before hospital discharge. Recent follow-up data were collected including echocardiographic evaluation (mean 30 ± 17.8 mo, range, 11 to 64). All patients were found to be in NYHA Class I or II. One patient with mitral repair was found to have moderate mitral insufficiency and NYHA Class II functional capacity.

**Table 2:** **Operative and postoperative data**

Variable	Mean ± SD or no
Operations	
MV replacement	11
MV re-replacement	4
Mitral plasty	5
Tricuspid plasty*	2
Cardiopulmonary bypass time	152 ± 28 min
Intensive care unit stay	2,4 ± 1 days
No. of red cell transfusion	2,8 ± 0,8
Inotropic support (early postoperative period)	5 patients
Inotropic support (beyond 24 h)	2 patients
IABP requirement	None
Postoperative stroke or neurologic dysfunction	None
Hospital stay	8,1 ± 2,9 days
Follow-up time	30 ± 17.8 mo

*Note that tricuspid plasty was performed as a concomitant procedure in two patients undergoing MVR. MV: Mitral valve; IABP: Intra-aortic balloon pump.

## Discussion

Our study involved 20 consecutive patients undergoing MV surgery after previous cardiac surgery/sternotomy via right thoracic minimally invasive access, deep hypothermic low flow CPB, and no aortic clamping. We have seen neither mortality nor neurologic dysfunction.

Advancing surgical techniques and blood conservation methodologies made reoperative cardiac surgery using repeat median sternotomy a common practice, with the resulting morbidity and mortality both low [16,17]. However, patients with previous CABG, especially ones with patent ITA grafts, remained a group posing dangers and necessitating caution — even for teams with a fading enthusiasm for right thoracotomy [16] — during a sternal reentry for MVR. Seeburger reported 6.6% mortality and 5.2% neurologic impairment rates in his article including 181 consecutive patients, where ventricular fibrillation was utilized in 140 of them at 28°C [8]. Casselmann experienced 3.8% mortality and 2.5% stroke rates in 80 consecutive similar cases undergoing endoscopic mitral and tricuspid surgery [11]. Even though these minimally invasive studies—as well as others [10,12,13] — report good results on a larger group of patients, such studies do not report the results of the exact same technique on their patients that we applied. Therefore, we hereby report our results, which strictly include video-assisted minithoracotomy, deep hypothermia, and low flow perfusion.

In Svenson’s study [18], comparing operative risks of a right thoracotomy versus sternal-reentry stroke rate was higher in right thoracotomy and right thoracotomy emerged as a risk factor for MVR. Several concerns raised by the authors included quality of mitral exposure and myocardial protection, and possible problems with peripheral arterial cannulation. In the same study, five out of the six strokes were found to be embolic in origin. Svenson [18] argued that the heart needs to be continuously fibrillating during the procedure in order to prevent an air embolism whose risk might be increased by any spontaneous conversion to regular cardiac contractions. Although it cannot be ruled out completely, if a spontaneous conversion to cardiac contractions occurs, this complication seems unlikely to us given the fact that left ventricular ejection would be into the low-resistance left atrial cavity because of the absence of a competent mitral valve, and presence of systemic high resistance above the competent aortic valve. Once the procedure is completed, however, the MV should be kept incompetent with a catheter passing through it. Experience shows that this complication is not a common finding [4-7], yet, it is not unseen [8]. Myocardial protection with fibrillatory arrest in the setting of deep hypothermia is satisfactory according to many others [4,6,7], our group among them. Svenson stressed in his article that right thoracotomy frequently afforded somewhat limited mitral exposure and a potentially reduced probability of MV repair [18]; we agree with this comment, as in only five (out of 16) patients were we able to perform a mitral repair. In our experience, this is partially attributable to the fact that the MV is positioned deeply in the thoracic cavity far from the operator. In addition, this technique involves deep hypothermia, and therefore a failed mitral repair on the postoperative TEE would necessitate an additional long period of CPB — including cooling and rewarming — to address the problem. This causes the surgeon to abandon the repair due to the reduced probability of a satisfactory outcome. Nonetheless, there are reports that the majority of the patients underwent mitral repair utilizing a similar technique [8,19]. In our series, four patients had re-replacement of the mitral valve. One patient had partial AVSD and three had rheumatic pathology; none of these eight was amenable for repair. In the remaining 12 patients (all with previous CABG), five (42% of the 12 patients, 25% of the total 20, with a mean EF of 47%) had MV repair. One of the last seven of our patients had a previous MVR. Of the remaining six of those seven, half of them (50%) had mitral repair, compared with 42% (the five of 12 mentioned above). We believe our increased confidence in this technique made us more liberal in MV repair, which accounts for our elevated use of the procedure. Our current concept on mitral repair when using this operative methodology is to keep it for the one who would less likely fail on postoperative TEE, based on the findings on preoperative TEE [15].

One of the major drawbacks of this procedure during MV intervention is a possible disturbance in the visibility of the MV due to back-bleeding. Murzi et al. used the Port-Access system with endoaortic balloon clamping and antegrade cardioplegia delivery to overcome this problem in patients with mild to moderate aortic insufficiency [10]. They mentioned using “additional doses of cardioplegia and deeper cooling to obtain satisfactory myocardial recovery since a significant portion of antegrade cardioplegia may go into the left ventricle”. However, in our view, this maneuver carries the risks of nonuniform cardioplegia delivery into the myocardium due to occlusion of the coronary graft ostia by the endoclamp, as well as cardioplegia wash-out by the functioning ITA, and may not be applicable at all in patients with previous CABG. Instead, short periods of very low CPB perfusion flow ― 0.2 to 0.5 liters/min ― can be helpful to improve visibility when bleeding is excessive. We try to restrict these occasions to the periods during which a more clear vision of MV annulus is absolutely necessary to put the stitch through. Those moments are kept as short as possible ― less than few minutes ― and immediately then blood flow is generally kept at or above 1.5 liters/min.

Patients with previous thoracotomies who have thick pleurae generally require extensive dissection on the lungs, which may implicate a heavy risk for postoperative pulmonary function [10,16]; for this reason, they too may not be suitable candidates for this operation. Since the initiation of our minimally invasive right thoracotomy procedures, we have seen patients with pleural thickness as well as pleural adhesion. We currently do not consider such patients for right thoracotomy so as to avoid the postoperative pulmonary dysfunction. Because adhesion is generally undetectable on preoperative imaging studies, during the operation, we prefer making a very small thoracostomy just big enough to pass a finger through before progressing to a minithoracotomy incision. If there is adhesion, we convert to sternotomy.

Therefore, in our view, considering the patients with isolated reoperative MV pathologies, a more-than-trivial aortic regurgitation and either pleural thickness or pleural adhesion are the conditions for which right thoracotomy for mitral surgery should not be contemplated.

Our results are comparable with results in other reports considering the postoperative blood loss, the number of transfused red cells, and the duration of intensive care unit and hospital stays [10,11]. So far, we are extremely pleased with our results, as no neurological complications occurred and low-output cardiac state was low with only 3 requiring inotropic support less than 24 hrs and 2 requiring support between 24–48 hrs. There was no profound low-output cardiac state as evidenced by the absence of mechanical circulatory support.

A 63-year-old patient with a 5.3 cm descending thoracic aneurysm died 6 mo postoperatively during an operation in another institution; his death was due to acute type III aortic dissection. He was scheduled for imaging studies to monitor the diameter of his descending aortic aneurysm. Preoperatively, he had hypertension, a mildly elevated blood creatinine level (1.6 mg/dL) and no known connective tissue disorder. Our strategy in patients with descending thoracic aneurysms is to consider endovascular repair once the aneurysm diameter reaches 5.5 cm.

We have both femoral and axillary arterial cannulations in our armamentarium, both with and without a Dacron sidearm. Concerning the 78-year-old woman who had thrombosis postoperatively in her femoral artery, even though it was observed preoperatively to be normal in CT study, the wall of that artery was found to be moderately calcified and she had known peripheral arterial disease distal to the femoral artery, so in retrospect we consider an axillary artery cannulation to be a better option in such circumstances.

Low incidence of inotropic need in our patients is consistent with the experience found in the literature that this technique provides very good myocardial protection [4-7]. Excluding the patients with peripheral arterial complications, the mean number of transfused red cell was 2.25 ± 0.7 (range, 2 to 4), and the mean hospital stay was 6.8 ± 0.8 days (range, 6 to 8).

The retrospective nature of this study and absence of data from a randomized control group (a sternal re-entry group) may restrict the accuracy of conclusions that can be drawn from the study.

## Conclusions

Based on our experience, we conclude that minimally invasive video-assisted MV surgery utilizing deep hypothermia, low-flow CPB without aortic clamping offers, more direct access to the MV and clinically, good myocardial protection, reproducible results, and relatively low rates of complications in patients with previous cardiac surgery via median sternotomy.

## Abbreviations

AVR: Aortic valve replacement
AVSD: Atrioventricular septal defect
CABG: Coronary artery bypass grafting
CHF: Congestive heart failure
CT: Computerized tomography
CXR: Chest X ray
NYHA: New York Heart Association
EF: Ejection fraction
IABP: Intra-aortic balloon pump
ITA: Internal thoracic artery MV, Mitral valve
MI: Mitral valve insufficiency
MS: Mitral valve stenosis
MVR: Mitral valve replacement
TEE: Transesophageal echocardiography
